# A theory of change for the inclusive education for learners with intellectual disabilities in South Africa

**DOI:** 10.4102/ajod.v14i0.1516

**Published:** 2025-08-13

**Authors:** Dane Isaacs, Precious Tirivanhu, Tlou M. Ramoroka, Mokhantšo Makoae, Noncedo Maphosho

**Affiliations:** 1 Desmond Tutu Centre for Religion and Social Justice, Faculty of Arts and Humanities, University of the Western Cape, Cape Town, South Africa; 2 Developmental, Capable and Ethical State Division, Human Sciences Research Council, Cape Town, South Africa; 3 Department of Development Planning and Management, Faculty of Management and Law, University of Limpopo, Limpopo, South Africa

**Keywords:** implementation of the education white paper 6, inclusive education, learners with intellectual disabilities, South Africa, theory of change

## Abstract

**Background:**

Since the implementation of the inclusive education policy in 2001 in South Africa, several milestones have been celebrated. Nevertheless, studies have reported the significant challenges in the implementation of the policy for learners with disabilities across schools in South Africa, which impede transformation.

**Objectives:**

Reflecting on key research insights from a recent study, this article argues for the development of a Theory of Change (ToC) as a road map or blueprint for implementing inclusive education for learners with intellectual disabilities in South Africa. A ToC is imperative for bridging the gap between policy implementation and outcomes.

**Method:**

A qualitative research design was adopted for the study. Data were collected through a desktop review, 12 key informant interviews and 10 focus group discussions with key role players and stakeholders in various positions in the education system (i.e. senior education specialists, special needs teachers, one education operations support officer, a principal, deputy principals and head of departments). The data collected were analysed thematically.

**Results:**

The findings of the study highlighted progress regarding government financial resources and also the various structural barriers impeding the implementation of inclusive education for learners with intellectual disabilities.

**Conclusion:**

This article encourages the continued development of theories of change to promote the effective implementation and ensure quality inclusive education for learners with disabilities in South African schools.

**Contribution:**

This article contributes a potential ToC to guide the effective implementation of inclusive education policy and practices for learners with intellectual disabilities in the South African schooling system.

## Introduction

There is growing recognition by policymakers, development practitioners and researchers on the potential role of theories of change in guiding the implementation of social policies and enhancing impact. In the context of educational transformation policies, a theory of change (ToC) plays multiple roles, assisting policymakers and key stakeholders to frame their transformation vision, guiding strategic choices, untangling complexities, guiding monitoring and evaluation and clarifying key change pathways towards the desired policy results (Clark 2021; INTRAC 2024; Salzano & Perrier 2024). A ToC has its historical roots in the discipline of theory-driven evaluation, which gained popularity in the 1990s (Goldsworthy 2021; Reinholz & Andrews 2020). Weiss (1995) popularised the phrase ‘theory of change’ through the efforts of the Roundtable on Community Change and the Aspen Institute. According to Weiss (1995:70), the ToC is an explicit process of thinking through and documenting how a programme or intervention is supposed to work, why it will work, who it will benefit (and in what way) and the conditions required for success. ToC is defined as a narrative:

[*D*]escribing the whole chain of influences (from outputs to impacts) of a project or programme up to its intended contribution to improving the lives of people in poverty, which is the aim of all our interventions. (Goodier, Apgar & Clark 2018:1)

Rogers (2014:1) defines a ToC as a graphic presentation that explains how activities are understood to produce a series of results that contribute to achieving the final intended impacts. ToC is also referred to as a ‘road map or blueprint for getting from here to there’ (Stachowiak 2013:2; Stein & Valters 2012:3).

The Education White Paper 6 (EWP6) (Special needs education: Building an inclusive education and training system) is a policy on inclusive education launched in South Africa in 2001. It promotes the education of all learners in South Africa. The policy also seeks to address all forms of learning barriers including transforming the curriculum, resource allocation and environments for learning, to ensure that all learners have access to equitable and inclusive quality basic education through public schools (Dalton, Mckenzie & Kahonde 2012). In 2014, the policy on Screening, Identification, Assessment and Support (SIAS) was further launched. The SIAS policy aims to provide learners with learning barriers with multi-layered support and encourages increased learner participation in mainstream schools (Makoae et al. 2023).

Research shows that several milestones in the form of structural, process and curriculum changes that the government introduced to bring about social change in the sector have been achieved (Du Plessis 2013; Mpu & Adu 2021). The changes recognised that exclusion from effective and equitable public education entailed the intersection of disability and institutional racism with child and youth vulnerabilities. Nevertheless, studies also report tenacious challenges in the implementation of the policy, particularly for learners with disabilities (Makoae et al. 2023). These challenges include the inadequate training of teachers, lack of parental support and involvement, poor resource allocation and curriculum challenges for learners with intellectual disabilities (Makoae et al. 2023; Mckenzie 2021).

The missing link between policy implementation and outcomes is a ToC. Presently, there is no explicit ToC that defines expected results, outlines the envisaged change pathways to attain results and outlines key assumptions about factors that lead or deter successful implementation of inclusive education in South Africa. Drawing on the key findings from a recent study (that aimed to evaluate the access to education support and services for children and youth with disabilities), the article presents a ToC towards the effective implementation of inclusive education for learners with intellectual disabilities in schools in South Africa.

A strong ToC can clarify any presumptions made about success of the implementation of the EWP6 with a focus on learners with intellectual disabilities and provide justification based on research and practice data currently available (Goldsworthy 2021; Kerr 2024; Reinholz & Andrews 2020). This can assist in evaluating the policy’s merits and potential to provide the desired results, as well as in delivering downstream programmes more successfully. It can also assist in informing the key stakeholders on the aims of the policy and providing justification for government or charitable expenditures. This enhances social accountability as societies have reached a stage where they require justification for inclusive education implementation (Loreman 2007). The value of a ToC for evaluation is another justification for its development as it guides development of indicators for inclusive education for learners with intellectual disabilities and allows monitoring and evaluation of the implementation process. Availability of this strategic tool in South Africa could assist the Department of Basic Education (DBE) and other stakeholders to systematically assess progress and plan for the provision of inclusive education for this group of learners. By explaining the strategy for transitioning from programme delivery to outcome achievement, one will recognise and gain insight into the collection of circumstances, actions and procedures that facilitate change (Goldsworthy 2021; Mintz & Roberts 2023).

In the next section, the methodology of the study is presented, and insights into the primary findings are also provided. These findings guided the development of a ToC which is presented in the proceeding section. This is followed by a discussion outlining a ToC in driving the implementation process for inclusive education for learners with intellectual disabilities in South Africa, before concluding.

## Research methods and design

### Study design

The broader study adopted a qualitative investigation in its evaluation of the access to education support and services for children and youth with disabilities. Data for the study were collected through a desktop review and empirical data collection. However, for the purpose of the article, the empirical aspects of the methodology only will be described.

### Recruitment and data collection

The empirical data for the study were collected through a series of Key Informant Interviews (KIIs) and Focus Group Discussions (FGDs). The study sought to get the input of relevant stakeholders on the strengths and challenges faced with regard to the implementation of the White Paper 6 for children and youth with disabilities in South Africa. Adopting a purposive sampling strategy, the study aimed to recruit a sample of key role players and stakeholders in the education sector (i.e. senior education officials, special needs teachers, class teachers, school-based support team [SBST] members, principals of institutions that provide education and support services for children and youth with disabilities [whether mainstream, full-service, special school or resource centre], principals of mainstream education schools [near the index of schools providing education for children with disabilities] and members of school-governing bodies).

Before commencing with the recruitment of participants, the research team sought to obtain ethical clearance from the nine provincial departments of education in South Africa. Once ethical clearance was obtained from the respective provincial departments of education, the research team searched the website of each provincial department to obtain a list of special needs schools, mainstream schools and full-service schools in these respective provinces. These schools were then contacted and informed about the study via telephone and/or email. The schools were included in the final sample of the research study on the following basis: (1) the obtaining of ethical approval from provincial departments; (2) their willingness to participate in the study; and (3) the availability of key role players and stakeholders in the education sector to participate in the study. The final number of schools included in the study were seven schools consisting of six special needs schools focused on intellectual disabilities and one primary full-service school in four education districts in three provinces in South Africa that is Mpumalanga, Limpopo and Gauteng.

The KIIs and FGDs were focused on assessing the progress, but also the challenges impeding the implementation of inclusive education for learners with disabilities in South Africa. Key informant interviews were conducted by researchers on the study. Twelve KIIs were conducted across the seven schools. It comprised five special needs teachers, two school psychologists, two senior education specialists, a principal and a deputy principal of a special needs school and an education operations support officer across the three provinces. The KIIs were guided by interview guides, and their duration was 12–80 min.

Because of the sensitive nature of the study, several participants were not comfortable engaging with the researchers in a one-on-one interview setting. They instead preferred group interaction because they did not want to be directly implicated. For this reason, participants were invited to participate in 10 FGDs. The questions in the KIIs informed the questions in the FGDs. The 10 FGDs were conducted across the seven schools by researchers on the project. The FGDs were divided according to two categories: The first category had participants who were a part of the management of special needs schools and had roles such as deputy principals, head of departments and a chief educational psychologist. A total of four FGDs were conducted under this group. The second category comprised six FGDs hosted with teachers. Five of these FGDs comprised special needs teachers, and one consisted of teachers in a full-service school. The duration of the FGDs was between 30 min and 80 min.

### Data analysis

The first author analysed 20 transcripts. These transcripts consisted of 12 KIIs and eight FGDs. These transcripts provided a comprehensive insight into the progress, but also the challenges impeding the implementation of inclusive education for learners with intellectual disabilities. Once these KIIs and FGDs were transcribed, the first author proceeded to analyse the data through inductive thematic analysis. The first step of the analysis involved importing the transcripts into Atlas ti.24. After reading and re-reading the transcripts, the first author proceeded to code the data. This brought about the first round of themes and sub-themes. These themes and subthemes were reviewed by the fourth author, who was also the principal investigator (PI) on the project from which the data for the article stemmed. The PI was asked to review the themes and sub-themes to ensure the accuracy of the data interpretation. The first author revised the analysis where necessary. This brought about the final themes and subthemes presented in the findings section further in the text.

### Ethical considerations

An application for full ethical approval was made to the Research Ethics Committee at the Human Sciences Research Council and ethics consent was received on the 06 December 2022 [REC 12/23/11/22]. Additionally, a second round of approvals was obtained from each provincial department of education, as previously mentioned. All procedures performed with human participants were in accordance with the research committee and with the 1964 Helsinki Declaration and its later amendments or comparable ethical standards. Written informed consent was obtained from all participants. The confidentiality of participants was protected by de-identifying participants’ responses and referring to them by a pseudonym in all future publications associated with the study.

## Results

As stated previously in this article, the data from KIIs and FGDs with participants in the study were categorised according to the following three themes and two subthemes: The first theme outlines the progress made with regard to inclusive education for learners with intellectual disabilities in South African schools. The second theme discusses the shortage of special needs schools as a barrier to inclusive education for learners with intellectual disabilities. The third and final theme, which is divided into two subthemes, describes the lack of physical and human resources as additional barriers towards the successful implementation of inclusive education for learners with intellectual disabilities in South African schools.

### Progress in inclusive education for learners with intellectual disabilities

The KIIs and FGDs revealed progress in terms of inclusive education for learners with intellectual disabilities. This was firstly with regard to adequate financial resources received from the provincial Department of Education for special needs schools, as highlighted in a KII with a deputy principal and in an FGD with the school management:

‘Our special schools get a lot of money from government. So, we get from the department, we get special allocation. So it’s quite a lot of money. I don’t know about the other schools and some of it it’s worked out per head in the special school. So, I don’t know how they work in mainstream schools and everything. So, our support funding will be worked out per learner and from that we can do different things to support the leaner.’ (Deputy principal, special needs school, KII 1)

‘I must admit it has increased, dramatically. In terms of resources, I think it’s managed by the school. We have enough funding to be able to buy resources. I don’t think the school was short of resources. We are able to purchase whatever the child needs. And definitely been taken care of. So, it has improved dramatically.’ (Special school management, P3, FGD 1)

The financial support received from the provincial Departments of Education also resulted in the modification and improved resource allocation to special needs schools:

‘Okay, what I’ve realised is that after the department have made the provision of giving them grants, when we visit the special schools, now they are all the same. They are resource centres, they’ve been modified, some were just having the stairs without the ramps. But as I’m speaking to you right now, we can see that things are happening.’ (Senior education specialist 1, district office, KII 2)

It is also important to highlight the fact that while the financial support of provincial departments was acknowledged, one special school in this study indicated actively raising funds towards the provision of resources for learners with intellectual disabilities in their schools. This was in the absence of receiving sufficient funding from the provincial department, as illustrated in the excerpt further in the text:

‘Our principal and SMT are really trying their best and we are really working hard. You know, we host a lot of functions. Our teachers do lots of fundraising to get money for resources. We are working hard on that. Some of the schools will envy us. They feel we are privileged to have such resources in our school. They ask where we get the money from. We started with nothing. It is our school’s own doing. We always raise funds all by ourselves. We would love if other people can get involved especially the department. We need more assistance from the department.’ (Special needs teacher, P3, FGD 1)

Nevertheless, the interview with the senior education specialist described earlier in the text secondly highlighted progress in terms of the good support received from the Provincial Department of Education in transitioning school institutions into full-service schools:

‘With that one I would say really the department is really on top of everything right now because the director himself at the level of the province, he’s not sending people to go and look for something. If there is something that must be done, and maybe they say they need to transform [*name of primary school deleted to protect the identity of the school*] primary school into a full-service school, he comes in here. He will just call us to say let’s meet at the school on this date and here is the agenda. Then he will come, explain the reason why they need to transform the school and then he will make it a point when he leaves there that relevant stakeholders are involved. He will also make it a point that he gives us the feedback to monitor and maybe even send us a monitoring tool to check the progress. If things are happening, then at the end of the day he will come to say according to the report that I’m having right now, then I can confirm that [*name of primary school deleted to protect the identity of the school*] is now a full-service school.’ (Senior education specialist 1, district office, KII 2)

Of equal importance, another senior education specialist, who participated in the study reported providing sufficient support to SBSTs. This support took the form of support meetings and workshops. These workshops were geared towards capacity-building:

‘Yeah, we do. Schools that do not have school-based support teams, we workshop them. We do like a workshop; you can even see our strategy under white paper 6. So, we train them about the roles of the school support team and then they’ll elect people who’ll be running the school support team and to indicate whether this team is functional or not functional.’ (Senior education specialist 2, district office, KII 3)

Subsequently, despite two participants outlining gaps in the functioning of SBSTs:

‘The SBST is there, but I can only say it’s only functional at a school level but not at a district level. Because here at school we have got our SBST team, which is the SMT and the support staff, the occupational therapist and the social workers. But if you can check, it’s not enough because we are not getting support from the circuit. We’re not getting support from the district. So, it’s only functional at a school level cause we are the ones who are faced with those challenges. So, we are the ones who has to deal with that.’ (Special needs teacher, P2, FGD 4)

‘Before I say no, we do not get visits from the department, from the district and wherever. They do not even know what we are doing here. They do not even know what they are supposed to support us with. We are really, I do not know what we, I will just say, but they neglect us. That is how we feel. They expect us to behave like mainstream and treat all the kids as education with no disabilities.’ (Special needs teacher, P6, FGD 2)

Several participants in this study reported well-functioning SBSTs – receiving enough support from this team within their respective schools:

‘Yes, we do have a functional SBSTs very functional one. I think a lot of schools do not have a functional SBSTs and that’s also where the support function comes in. I think that was being a big part of the district, getting SBSTs to be functional. So, everyone is part of our SBSTs. We all have a role in it and we support our learners. We also meet once a week and will talk about new learners that maybe came in battling and we discuss the learners that are maybe starting now with the subject.’ (Full-service teacher, P3, FGD 1)

Lastly, progress was observed through the effective and increased implementation of the SIAS policy in schools:

‘I won’t speak on behalf of others, but for us, I think, we are doing a very good job. Each year, the learners are screened, Grade *R*-one, seven and eight. There is learner identification that is done in the beginning of the year. Assessments are also done, and we have got the inclusion of the students. I know all the districts do that and we distribute them separately. The assessment of the identified learners in this area is done.’ (Education operations support officer, district office, KII 4)

The effective and increased implementation of the SIAS policy was also reported at the Foundation phase. The identifying and profiling of children with disabilities and learning difficulties happened early in the schooling career of learners with intellectual disabilities:

‘As in terms of the White Paper 6, the objectives of the implementation of SIAS in the foundation is ongoing. Let me be honest with you, things are happening in the foundation. Every now and then when we are having workshops or we are having the meeting. We never ever have such kind of occasion without reminding them about the implementation of SIAS and reminding them about the adherence to the White Paper 6.’ (Senior education specialist 1, district office, KII 2)

As highlighted in the excerpt earlier in the text and the excerpt further in the text, the successful implementation of the SIAS policy was linked to increased awareness about the policy, which was argued to transform the inequities characteristic of the education system and led to schools embracing protocols that support differentiated learning:

‘Even at our education summits in the district, we make sure that inclusive education is a song that is sung by everyone. Even now, we used to receive few concessions from schools located in the informal settlements. Concessions were only made by the Model C schools but today they know the protocols of concessions and accommodations and very soon this district will be far …’ (Senior education specialist 2, district office, KII 3)

### Shortage of special needs schools

Despite the positive progress reported in the previous theme, participants highlighted the various challenges and gaps impacting on the successful implementation of inclusive education for learners with intellectual disabilities. One such challenge is the shortage of special needs schools, which participants perceived as essential for the implementation of inclusive education for learners with intellectual disabilities in South Africa. Special needs schools were identified as centres where these learners can receive the support they require:

‘I would say that I’m not a politician. Because I think we are only trying to solve this case and I know each and every school will have a small or big school’s support. As I said, we do the screening yearly, so those grades are mentioned but this is the individual support. We want to identify learners because we have such learners in each and every school. So, we still need more schools and schools with special classes. We need more of those because we only have 12 now.’ (Education operations support officer, district office, KII 4)

‘Our learners are living with several intellectual disabilities in such a way that they do not qualify for mainstream type education, basically, to do the national curriculum statement. They are doing what we call D-CAPs (Differentiated CAPs). We have aligned it in such a way that it will suit their needs.’ (Principal, special needs school, KII 5)

At the same time, the principal of a special needs school in the excerpt cited earlier in the text described special needs schools as safe and protective space for learners with intellectual disabilities:

‘The people are labelling them. And that is why if you can check on our children, they enjoy themselves much when they are with us than when they are with their community or with their parents at home. It is because they have realised that at school we get more acceptance and attention than at home. At home they are called names because of their disabilities. So if you can come to our school maybe on Monday or immediately after the school open, you will realise the joy on our children. But on Friday, or the last day of the term, you will realise that the children are not happy. So, obviously they start thinking to say that where we are going now, we are going to be isolated. Some of them, they are locked up in the room.’ (Principal, special needs school, KII 5)

However, existing special needs schools in the study were described as overcrowded, having long waiting lists and not having the necessary staff to handle the influx of learners:

‘We are overwhelmed in the sense that there is still a long waiting list. We are trying our best. Just recently we have built new classrooms. We have started this whole section to be an autism unit. So, the school really tries to do a lot to try and relieve the problems that the Department of Education is having in terms of space for learners, but still, it is not enough. There are not enough special needs schools. I personally feel they are weakening our schools just by putting more children and more children in because we do not always get more support staff and we try to manage with that.’ (School psychologist, special needs school, KII 6)

The overcrowding of special needs schools resulted in the overcrowding of classrooms. In one of the FGDs hosted with special needs teachers, one teacher described not being able to provide learners with the necessary attention and support they require because of the size of classes:

‘Some schools struggle with overcrowding in classrooms and then there is only one teacher in the class. It is difficult to get to know every single learner properly who is sitting in front of you. Then problems are dismissed, If there is a smaller class, you get to know the kids one-on-one and identify certain problems. It is easier to screen a kid, identify and send them to the right school where they can get the necessary help. But we see it only when the kid is already 16 or 17 years old and then they can only come here, it is already too late.’ (Special needs teacher, P5, FGD 2)

In another FGD with teachers from a full-service school, one teacher expressed concerns about the waiting lists into special needs schools typically resulting in learners with intellectual disabilities having to remain in full-service schools:

‘The waiting list of enrollment starts when you start running special schools and vocational schools. So, we keep them here. They don’t go to another school unless they get placement in [*name of school deleted to protect the identity of the school*]. Our special schools are really overpopulated at this stage. So, we had a few learners here that were autistic that was on the autistic spectrum, nonverbal. And they only got placed at the end of last year, beginning of this year.’ (Full-service teacher, P3, FGD 1)

This teacher described the full-service school she taught at as overpopulated. As a result, she was unable to provide learners the necessary support they require:

‘I think the amount in a classroom is the biggest challenge for one teacher without an assistant to accommodate 40 learners. And when I say the learners need support, it is not group support. We have learners that cannot read and write. We have learners that is autistic that cannot speak. We have to accommodate them as well. So, in order for it to be more effective, I think with more support for the teachers, less learners in a classroom, it can be effective.’ (Full-service teacher, P3, FGD 1)

An interview with a school psychologist at a special needs school provided additional insight into the gaps and barriers surrounding special needs schools. This included special needs schools being kilometres away for both learners and their parents:

‘I know about that school, but that is actually the only school that I am aware of and was changed. We know that there must be one in another city. We see many children coming from there to our school. So, there is a big need for our type of school there. This creates massive transport issues to get all those children from there to us here. There are not enough buses. There is a bus waiting list. Sometimes the child gets space in a school, but not on the bus, and the parents cannot bring the child to school because they do not have the means to take the child every day to the school. That is a challenge.’ (School psychologist, special needs school, KII 6)

The interview with this psychologist also brought to the fore the poor documentation of learner information at schools and a lack of efficiency in the district offices which have been found to further delay learners from gaining access to special needs schools:

‘It must go through the Department of Education again. So, there is a problem because during this time, the child will be sitting at home. He cannot just go to a new school. We cannot just pick up the phone and say, listen, this child was with us. We will fax or send documents. All these children must go through the district office again to facilitate the placement. There is not enough manpower at the district offices to do these things. Some of our children and parents remain for two or three years on those waiting lists before they get placements. So, the whole paperwork thing is also not done effectively. For example, you have hard copies of a file now, someone must scan it and email to someone then at the next school it must be printed out. Some of the schools do not always have the resources to trace all those papers. If the file is missing, then there is no way the child’s information will be traced again. So, then it must be started from scratch.’ (School psychologist, special needs school, KII 6)

Finally, the interview with the school psychologist, alongside other participants in KIIs and FGDs discussed how parent denial and the stigma associated with special and vocational schools, contributed to learners with intellectual disabilities remaining on waiting lists to enter special needs schools:

‘I think education of parents is a big thing because a lot of the children that do not get placed, it is parents that say no, my child is staying where he or she is. Not understanding why, the child needs to move or why it could be best for the child to move. One mom that I know told us she thinks we must put her child in an English class because he was in African school, and he did not do well. No, your child did not do well because he was in an African school. He was diagnosed with severe intellectual disability. He would not have done well in an African or in English mainstream school, so parents’ and community education is quite important.’ (Deputy principal, special needs school, KII 1)

‘Unfortunately, we do experience parents that are not always involved. Parents that do not always understand that this environment is not the best place for their child. And that because of the stigma that vocational schools and special schools have. Sometimes, they do not want them to go that route. So, they will progress up until they go to high school. And then you sit with a child that’s 15, 16 that needs to be enrolled in high school and eventually they drop-out.’ (Full-service teacher, P3, FGD 1)

Such action also resulted in parents not exploring suitable pathways of education for learners, which at times resulted in learners commonly dropping out of school, as highlighted in the second excerpt above.

### Lack of physical and human resources

In addition to the shortage of special needs schools, participants identified other challenges when implementing inclusive education for learners with intellectual disabilities. This included: (1) physical resources and (2) human resources.

#### Physical resources

Mainstream schools for learners were described as not accessible to learners with physical disabilities.

‘Yeah, what we also need is to make space for the physically disabled. Because we don’t have enough schools for that. The ramps in the mainstream. The classroom, they need to be accessible, almost all are not accessible for learners with physical disabilities because the townships are struggling. So, we are saying that the facilities there need to be refurbished.’ (Education operations support officer, district office, KII 4)

Similarly, a teacher in the second FGD for special needs teachers, highlighted the shortage of infrastructure for the diverse needs of learners with intellectual disabilities:

‘I think it is very difficult for a school to provide facilities for all the types of disabilities that they have.’ (Special needs teacher, P4, FGD 2)

Moreover, curriculum design was identified as another shortcoming in institutional arrangements for the delivery of inclusive education for learners with intellectual disabilities. The participants further in the text described the curriculum used in their respective schools as burdensome to teachers, and not adapted for the differentiation of all learners’ level of intellect:

‘The content is too much. The assessment is too much as well. There is so much pressure on the teachers that sometimes you are also busy with administration that you do not always have time to do the proper teaching and these kids are hands on hands.’ (Special school management, P4, FGD 2)

‘Yes, they do. The only thing available is just the curriculum for the little ones in foundation phase. They definitely do get support, but the curriculum is so difficult for our children, and it is mostly more appropriate for mainstream children. Most of the children in my class, struggle extremely with the curriculum and then there are children who cannot do it at all so, I think we do get support but where more support is needed is for the curriculum adaptation and the differentiation for all the children’s level of intellect.’ (Teacher 1, special needs school, KII 7)

This participant in the first excerpt, alongside other participants in the study, reported not receiving the necessary training and support from the department of education to effectively implement the curriculum:

‘We send letters every year to the department because we are struggling with the curriculum because it is not fit for our children, and they do nothing. Teachers are alone. There is no help, no support. If we don’t figure it out for yourself, we are not going to make it. The teachers from other schools come here all the time because they do not know what to do. They want us to train them, but then the department has got people that supposed to train them.’ (Special school management, P4, FGD 2)

‘We never get any curriculum advisors that visit our special school neighborhood. We only see our circuit manager; he comes regularly, and he is very supportive. We are now drafting number five for the profound curriculum. It is ridiculous. We had one training, so in five years. I think we are the only school actually who successfully running the profound curriculum.’ (Special school management, P4, FGD 2)

Furthermore, one teacher in this study expressed concern about the inadequacy of programmes in special needs schools to provide learners with intellectual disabilities with the necessary skills to enter the labour market:

‘What is going to happen to those children after school? What support do they get after leaving school? Like after school programmes to get jobs. It is actually so hard but most of the times the children, when they leave school, there are small programmes but not all the children can do it. Then what happens to those children, especially if their parents die, not being able to take care of themselves? So, I think they do get support from the district, but it is more for when they leave school, what happens to those children?’ (Teacher 2, special needs school, KII 8)

Adding to the discussion earlier in the text, a principal in this study critiqued the White Paper 6 for not providing learners with intellectual disabilities with formal certification when exiting the school system. The school where this principal is based seeks to provide learners with intellectual disabilities with vocational skills. However, as shown in the quote further in the text, these vocational skills are not recognised in the labour market without relevant accreditation. This was argued to often lead to unemployment among learners with intellectual disabilities:

‘For instance, there are those who are doing the car wash. When they leave here, they go and do the car wash. There are those who are doing manicure and pedicure. The nails, the hair dressing and whatever. So, at least when they leave school, they can be able to go and open their salon. And be able to get a living out of it. But when they leave schools, they will only be having a portfolio. We are still struggling with the white paper 6 on SITA. Because we are hoping that SITA maybe one day might come and accredit the skills that these learners are doing. But they do not have any certificate. So how are they going to get employed?’ (Principal, special needs school, KII 5)

‘So, if that work can be taken to maybe SITA and whatever to say that let us accredit this. But for now, when they go to any company around to say that we are looking for a job, they want the certificate. They want the paper. The paper is not there. But we know very well that if we can give that child an opportunity, he will perform.’ (Principal, special needs school, KII 5)

The principal called on the SITA to accredit the vocational skills of learners with intellectual disabilities so that they can obtain employment.

#### Human resources

The shortage of school psychologists was recognised as another challenge for inclusive education involving learners with disabilities:

‘I think they need more psychologists at school level to do the assessments for that particular school, so that it doesn’t have backlogs. Because if you’ve got more people, if every special school has one or two psychologists, you could do bulk of the assessment, instead of having maybe five psychologists dealing with 50 schools, and having to get through that big caseload.’ (Special school management, P5, FGD 3)

The participant above called for the employment of more school psychologists. He explained how the lack of school psychologists had resulted in the delayed assessment of learners with intellectual disabilities.

In a similar vein, participants highlighted a shortage of caregivers for learners with severe intellectual disabilities in the classroom:

‘I think it’s finance because like I was saying, the policies say that they will have support staff, when they have more learners with disability. There are many learners that are using pampers, others are on wheelchairs. So, it is difficult to accommodate them when they don’t have teachers. The teachers there is only one in the classroom. The learners in the wheelchair, you need somebody who will help them to go to the toilet. If they had the caregiver, they could assist.’ (Senior education specialist 2, district office, KII 3)

‘They cannot dress and undress themselves. The school appointed a few ladies to work as caregivers. For me, this is something that the Department of Education needs to look into. There must be specific posts established for them because now they work on yearly contracts and their salaries are low. We depend on them. I think there must be specific posts for those types of services. I really think the department is in a way neglecting that. Some of our learners intellectually, they are low functioning, they need someone that can take care of them.’ (School psychologist, special needs school, KII 6)

The participants in the second excerpt accordingly called provincial departments to provide permanent employment for caregivers to provide adequate support and care for learners with severe intellectual disabilities in the classroom.

Furthermore, although participants have acknowledged progress in the training of teachers in special needs education, they highlighted gaps in the practical experience in the tertiary education of teachers:

‘So, I feel in theory, it is easier for us because we know this is what a special needs child is, but I do feel we need to get more practice, more physical practice. Many a times what happens is you get training, online training, everything from the government, which is nice and then you acquire the knowledge but then you go into class and put that knowledge into practice is difficult. So, I think if we can get opportunities where we can go to other special needs schools, see what their teachers are doing, how they differ from us, and they also come to us then we can learn from each other. I think that would be much easier and better and also because sometimes as a special needs educator you do feel that you are not always doing exactly what you should be doing, and you are not sure if you are fulfilling the needs of the learners. So, if you can go to other places, you will see that you are actually doing a good job and it motivates you.’ (Teacher 2, special needs school, KII 8)

‘It is not like 100% everyone has the skill. With the children, you must have patience and people are not the same. There are those who are harsh and those who are quick to take action and stuff like that. But with this kind of learners, you must really have patience and be compassionate because conflicts are always there.’ (Teacher 3, special needs school, KII 9)

In the fourth focus group with management, one participant additionally highlighted the shortage of teachers to teach practical subjects:

‘We do not have technical teachers. Not having technical teachers is a huge problem. They do not want to appoint them. They do not have proper education degrees. Yeah, but they are on the technical side. Teachers must be appointed and work in school. We do not have teachers that can teach practical subjects, technical subjects.’ (Special school management, P1, FGD 4)

## Discussion

**FIGURE 1-A1:**
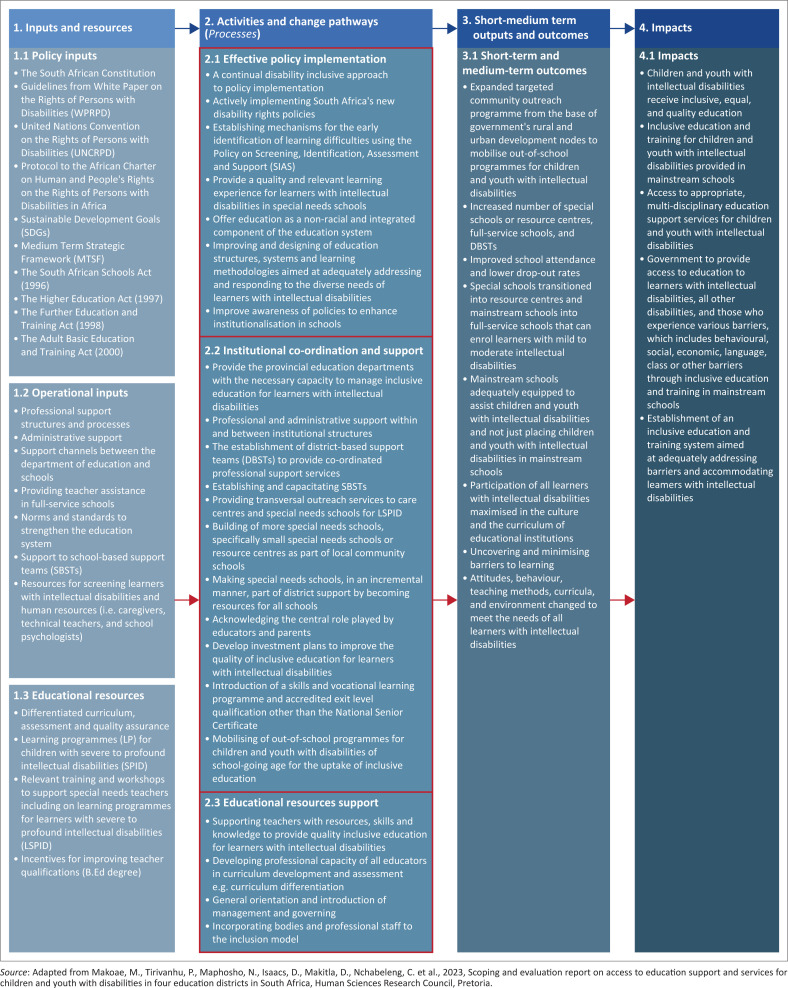

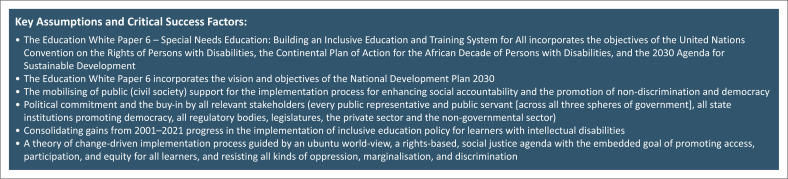
A theory of change for the inclusive education for learners with intellectual disabilities in South Africa.

Guided by findings from the empirical evidence (outlined in the preceding section), a ToC was developed to provide conceptual guidance for future ToC-driven implementation of the EWP6 for learners with intellectual disabilities in South Africa. According to De Silva et al. (2014) and Wu et al. (2024), a ToC-driven implementation process strengthens the development of interventions by providing a framework for enhanced stakeholder engagement and explicitly designing policies or programmes that are embedded in the local context. A ToC also improves strategic planning or programme and policy planning and evaluation of interventions through a comprehensive set of indicators to evaluate various causal pathway stages through which an intervention achieves impact (Hiltensperger et al. 2024; Rogers 2014). Figure 1-A1 in Appendix 1 outlines the ToC towards the future implementation of the EWP6 for learners with intellectual disabilities in South Africa.

The resultant ToC makes several key assumptions for successful implementation of EWP6 for learners with intellectual disabilities. According to Mayne (2023), assumptions in a ToC are the causal connections, events and conditions that need to be realised for the intervention to work. They consider the context in which an intervention operates (Vanderkruik & McPherson 2017). The key assumptions for the effective implementation of the EWP6 for learners with intellectual disabilities in South Africa, include the need to incorporate objectives of the United Nations Convention on the Rights of Persons with Disabilities, the Continental Plan of Action for the African Decade of Persons with Disabilities and the 2030 Agenda for Sustainable Development. These provide the global framework, and benchmarks to define the implementation process.

Similarly, another assumption is that the implementation of the EWP6 for learners with intellectual disabilities should be guided by the vision and objectives of the National Development Plan 2030 in South Africa. This involves the mobilising of public (civil society) support for the implementation process for enhancing social accountability and the promotion of non-discrimination and democracy. There is also a need for political commitment, the buy-in by all relevant stakeholders (every public representative and public servant [across all three spheres of government], all state institutions promoting democracy, all regulatory bodies, legislatures, the private sector and the non-governmental sector) and the consolidation of gains from 2001 to 2021 progress in the implementation of inclusive education policy for learners with intellectual disabilities. In addition, the ToC-driven implementation process should be guided by an ‘ubuntu world-view, a rights-based, social justice agenda with the embedded goal of promoting access, participation, and equity for all learners, and resisting all kinds of oppression, marginalisation, and discrimination’ (Muthukrishna & Engelbrecht 2022:66).

A ToC is made up of a results chain. The results chain of the ToC includes inputs and resources, activities, change pathways, short-medium term outputs and outcomes and the envisaged impacts. The United States Agency for International Development (2016) defines a results chain as:

[*A*] type of logic model that displays the relationships between what a programme intends to do and the changes and results it hopes to accomplish to achieve its programme purpose. (p. 9)

Through a sequence of anticipated intermediate outcomes, it illustrates the presumptive causal relationship between an intervention and desired impacts (Foundations of Success 2009; Hiltensperger et al. 2024; Wu et al. 2024). Results chains are important in clearly specifying assumptions behind an action and positioning implementors to develop relevant objectives and indicators to monitor and evaluate whether actions contribute to the intended impact (Margoluis et al. 2013; Salzano & Perrier 2024). According to Zwart (2017) and Salzano and Perrier (2024), result chains provide a framework for results-based management through linking results to goals, ensuring results-based management approaches are fit for purpose and linking results and performance information for better delivery.

The inputs and resources for the effective implementation of the EWP6 for learners with intellectual disabilities are categorised into policy, operational and educational resources. The key policy inputs include the South African Constitution, guidelines from the White Paper on the Rights of Persons with Disabilities, United Nations Convention on the Rights of Persons with Disabilities, Protocol to the African Charter on Human and People’s Rights on the Rights of Persons with Disabilities in Africa, Sustainable Development Goals, Medium Term Strategic Framework, *the South African Schools Act* (1996), the *Higher Education Act* (1997), the *Further Education and Training Act* (1998) and the *Adult Basic Education and Training Act* (2000). The EWP6 has not been reviewed since the Committee on the Rights of Persons with Disabilities issued the General Comment 4 to uphold inclusive education that emphasises participation of children with disabilities in mainstream education as a fundamental human right of all learners (United Nations Convention on the Rights of Persons with Disabilities Section 10a). The above provides the fundamental policy and legislative frameworks for the effective implementation of the EWP6 for learners with intellectual disabilities.

Operational inputs include professional support structures and processes; administrative support, support channels between the department of education and schools, providing teacher assistance in full-service schools, and norms and standards to strengthen the education system, among others. Other inputs highlighted in KIIs and FDGs include support to SBSTs (e.g. through capacity building workshops), resources for screening learners and human resources (including caregivers, technical teachers and school psychologists). Educational resources include differentiated curriculum, assessment and quality assurance; and learning programmes for children with severe to profound intellectual disabilities. The gap in the training of special needs teachers (as highlighted in the KIIs) additionally emphasises the need for relevant training and workshops to support special needs teachers including on learning programmes for severe to profound intellectual disabilities; and incentives for improving teacher qualifications (B.Ed. degree) and continuous training.

These three categories of inputs and resources feed into complementary activities and change pathways. These are: (1) effective policy implementation; (2) institutional co-ordination and support; and (3) educational resources support. With regards to effective policy implementation, key activities and change pathways include a continual disability-inclusive approach to policy implementation, actively implementing South Africa’s new disability rights policies; establishing mechanisms for the early identification of learning difficulties using the SIAS policy; provide a quality and relevant learning experience for learners with intellectual disabilities in special needs schools; and offering education as a non-racial and integrated component of the education system. Additionally, focus will be placed on improving and designing of education structures, systems and learning methodologies aimed at adequately addressing and responding to the diverse needs of learners with intellectual disabilities. The KIIs and FGDs also highlighted the need to improve awareness of policies to enhance institutionalisation in schools.

The activities and change pathways under institutional coordination and support include: Providing the provincial education departments with the necessary capacity to manage inclusive education for learners with intellectual disabilities. It will also include the professional and administrative support within and between institutional structures; the establishment of district-based support teams (DBSTs) to provide co-ordinated professional support services; the establishing and capacitating of SBSTs; and providing transversal outreach services to care centres and special needs schools for learners with severe to profound intellectual disabilities. In light of the shortage of special needs schools (as outlined in the KIIs and FGDs), concerted efforts should be made to build more special needs schools to counter long waiting lists and to ensure learners with intellectual disabilities obtain the support they require. The findings of the study showed that special needs schools are often long distances from families. Echoing the sentiment of Muthukrishna and Engelbrecht (2022), there is a need for the building of small special needs schools as part of local community schools to make it more accessible to families and local communities. Special needs schools, in an incremental manner, should also form part of district support by becoming resources for all schools. Focus will further be placed on acknowledging the central role played by educators and parents; the development of investment plans to improve the quality of inclusive education for learners with intellectual disabilities; and the introduction of a skills and vocational learning programme and accredited exit level qualification other than the National Senior Certificate. In a similar vein, there will be the mobilising of out-of-school programmes for children and youth with disabilities of school-going age for the uptake of inclusive education. These actions seek to address the unemployment crisis presently affecting learners with intellectual disabilities, as highlighted in the KIIs of the study.

Under educational resources support, key activities and change pathways include: supporting teachers with resources, skills and knowledge to provide quality inclusive education for learners with intellectual disabilities. This includes developing professional capacity of all educators in curriculum development and assessment, for example, curriculum differentiation. It further involves the general orientation and introduction of management and governing; and incorporating bodies and professional staff to the inclusion model.

The activities and change pathways will lead to short medium-term outputs and outcomes. These include an expanded targeted community outreach programme from the base of government’s rural and urban development nodes to mobilise out-of-school programmes for children and youth with intellectual disabilities; increased number of special schools or resource centres, full-service schools and DBSTs. Moreover, the activities and change pathways would also improve school attendance and lower drop-out rates; transition special needs schools into resource centres and mainstream schools into full-service schools that can enrol learners with mild to moderate intellectual disabilities. Despite data not being collected from mainstream schools, mainstream schools are an essential part of the implementation of inclusive education for learners with intellectual disabilities. Therefore, another outcome is that mainstream schools are being equipped to adequately assist children and youth with intellectual disabilities and not just placing children with intellectual disabilities in mainstream schools. It is important that participation of all learners with intellectual disabilities is maximised in the culture and the curriculum of educational institutions. Likewise, there needs to be an uncovering and minimising of barriers to learning; a change in attitudes, behaviour, teaching methods, curricula, and environment to meet the needs of all learners with intellectual disabilities in South Africa.

Five major impacts are envisaged from the ToC-driven implementation of EWP6 for learners with intellectual disabilities in South Africa. These are: (1) children and youth with intellectual disabilities receive inclusive, equal, and quality education; (2) inclusive education and training for children and youth with intellectual disabilities provided in mainstream schools; (3) access to appropriate, multi-disciplinary education support services for children and youth with intellectual disabilities; (4) government to provide access to education to learners with intellectual disabilities, all other disabilities, and those who encounter various barriers to learning, which includes behavioural, social, economic, language, class or other barriers through inclusive education and training in mainstream schools; and (5) establishment of an inclusive education and training system aimed at adequately addressing barriers and accommodating learners with intellectual disabilities.

## Conclusion

South Africa’s policy on inclusive education was implemented from 2001. Although this implementation process achieved several milestones in the form of structural, process, institutional arrangements and curriculum changes, challenges remain in the implementation of the policy for learners with intellectual disabilities. Such challenges include lack of special needs schools, inadequate curriculum design and lack of resources (both physical and human resources) to support learners with intellectual disabilities. This research attributed such implementation failure to the lack of an explicit ToC. This research developed a ToC to provide a conceptual view for the implementation of the policy for learners with intellectual disabilities.

The ToC outlines five key envisaged impacts and defines the results chain with explicit inputs and resources, activities, change pathways, short-medium term outputs and outcomes. The inputs and resources are categorised into policy, operational and educational resources. These feed into complementary activities and change pathways (effective policy implementation, institutional co-ordination and support and educational resources support). The ToC will allow identification of potential risks and challenges associated with the policy implementation process and allow development of mitigation measures. It will further provide a framework for measuring and evaluating the impact of the policy implementation process. While this ToC presents a key step in rationalising the policy implementation process, it needs to be verified by key stakeholders to ensure stakeholder buy-in. In addition, there is a need to develop relevant institutional arrangements, operational modalities and matrices (including monitoring and evaluation frameworks) to ensure effective implementation of the policy for learners with intellectual disabilities through a ToC driven process.

Beyond learners with intellectual disabilities, the continued development of theories of change is strongly encouraged to guide the effective implementation of inclusive education for learners with other disabilities in South African schools. Learners with disabilities, in South Africa, should have access to quality education, and also an education system that effectively promotes and responds to their individual needs and rights.
